# AI-Generated Avatar Videos for Postoperative Patient Education Among Health Care Workers: Pilot Randomized Controlled Trial

**DOI:** 10.2196/89277

**Published:** 2026-05-27

**Authors:** Syed Ali Haider, Srinivasagam Prabha, Cesar Abraham Gomez-Cabello, Ariana Genovese, Bernardo G Collaco, Nadia Wood, James London, Sanjay Bagaria, Mark A Lifson, Cui Tao, Antonio Jorge Forte

**Affiliations:** 1 Department of Surgery, Division of Plastic Surgery, Mayo Clinic in Florida, 4500 San Pablo Road, Jacksonville, FL, 32224, United States, 1 904-953-2073; 2 Department of Radiology, AI IT, Mayo Clinic, Rochester, MN, United States; 3 Department of Surgery, Mayo Clinic in Florida, Jacksonville, FL, United States; 4 Division of Surgical Oncology, Mayo Clinic, Jacksonville, FL, United States; 5 Center for Digital Health, Mayo Clinic, Rochester, MN, United States; 6 Department of Artificial Intelligence and Informatics, Mayo Clinic in Florida, Jacksonville, FL, United States

**Keywords:** artificial intelligence, AI, text to video, generative artificial intelligence, generative AI, postoperative instructions, patient education

## Abstract

**Background:**

Effective postoperative communication is vital for patient recovery, yet traditional text-based discharge instructions often lead to poor comprehension and adherence, particularly among patients with limited health literacy. Although educational videos improve understanding and retention, their widespread use has been hampered by high production costs. Generative artificial intelligence (AI) offers a scalable solution for creating engaging video content.

**Objective:**

The primary objective of this pilot study was to assess the feasibility of creating and deploying AI-generated, avatar-led videos for postoperative instruction delivery. Secondary objectives included comparing knowledge retention, engagement, perceived clarity, and user experience between AI-generated video and traditional text-based handout formats among health care workers.

**Methods:**

In this randomized pilot study, 38 health care worker volunteers were recruited as a convenience sample to pilot-test the intervention before patient implementation. Participants were assigned to either a text handout group (n=19, 50%) or an AI-generated video group (n=19, 50%). Both groups received information on 10 common postoperative topics. The primary outcome was objective knowledge, assessed via a 10-item quiz. Secondary outcomes, measured through surveys with 5-point Likert scales, included engagement time, subjective engagement, perceived clarity, usefulness, confidence in understanding, and information retention. Qualitative feedback was also collected.

**Results:**

Objective knowledge quiz scores did not differ significantly between groups (mean 8.89, SD 1.20 for the AI-generated video group vs mean 8.21, SD 1.78 for the text handout group; *P*=.17; Cohen *d*=0.45). Participants in the AI-generated video group demonstrated significantly higher engagement time (mean 15.11, SD 7.78 minutes vs mean 8.84, SD 4.03 minutes; *P*=.004; Cohen *d*=1.04). They also rated instructions as significantly clearer (mean 4.63, SD 0.50 vs mean 4.00, SD 0.82; *P*=.007; Cohen *d*=0.93), more engaging (mean 4.05, SD 0.78 vs mean 3.32, SD 1.00; *P*=.02; Cohen *d*=0.81), and more effective for retention (mean 4.42, SD 0.84 vs mean 3.37, SD 0.68; *P*<.001; Cohen *d*=1.38). Qualitative feedback highlighted the engaging nature of AI-generated videos but noted areas for avatar refinement.

**Conclusions:**

In this pilot study with health care workers, AI-generated avatar videos did not improve objective knowledge scores but significantly enhanced engagement, perceived retention and perceived clarity (Cohen *d*=0.81‐1.38). Future studies in actual patient populations with diverse health literacy levels are needed to determine whether these engagement advantages translate into improved knowledge outcomes.

## Introduction

Effective postoperative communication at hospital discharge is critical, yet many patients leave with written instructions they cannot fully understand or remember. Approximately 90 million adults in the United States have limited health literacy [1]. Patients with lower educational attainment may struggle to recall instructions, leading to medication errors, missed follow-ups, and adverse outcomes [2-5]. Poor adherence to discharge instructions contributes to preventable readmissions, patient dissatisfaction, and increased health care costs [6-8]. Traditional instruction methods, such as written materials and brief verbal explanations, rarely deliver information in a memorable or actionable way [9-11].

Educational videos offer a compelling solution [12,13]. They simplify complex information, support diverse learning styles, and improve comprehension and retention—especially when paired with written materials [12]. Videos can demonstrate procedures visually, feature presenters who create a sense of social presence, accommodate varying literacy levels through combined auditory and visual information, and offer the potential for multilingual messaging. However, high production costs, long turnaround times, and equipment requirements have limited their widespread use.

Generative artificial intelligence (AI) enables the rapid, automated creation of video content [13]. Platforms such as OpenAI’s Sora and Google DeepMind’s Veo can generate high-quality, coherent video content directly from simple text prompts [14], with applications proposed across medical domains, including surgical training, public health education, and patient-specific educational content [15-17]. A particularly compelling feature is AI-generated avatars—photorealistic digital humans who deliver content in a natural manner.

According to Mayer’s cognitive theory of multimedia learning [18], combining verbal and visual information enhances understanding by engaging dual channels in the brain. Visible instructors create social presence, increasing motivation and attention [19]. A systematic review found that videos featuring visible instructors consistently improved learning outcomes [20]. Early evidence in health care supports this: a virtual nurse avatar for discharge instructions was well received, with 85% of patients liking the character and 86.4% of nurses believing it would aid education [21]. AI-generated avatars have been explored for HIV education, heart disease knowledge dissemination, and ileostomy education [22-24], and more recently, interactive physician avatars for postoperative patient education have demonstrated high usability and trust among surgical patients [25].

By assessing both objective understanding and subjective experience, this study aims to examine the feasibility and user experience of AI-generated video as a medium for patient education at hospital discharge. If effective, this approach could help reduce disparities in postoperative care by providing patients with clearer, more accessible discharge instructions.

## Methods

### Study Design and Participants

The primary objective was to assess the feasibility of creating and deploying AI-generated avatar videos for postoperative instruction. The primary outcome was the difference in knowledge quiz scores between groups. Secondary outcomes included engagement time, subjective engagement, perceived clarity, usefulness, confidence in understanding, information retention, and preference for format. Qualitative feedback was also collected through open-ended questions.

We conducted a pilot study to compare traditional text-based instructions with AI-generated video instructions for postoperative care education. A total of 38 adult health care worker volunteers were recruited from a hospital community (faculty, staff, researchers, and students). This convenience sample of health care workers was chosen (1) to pilot-test the intervention in a controlled setting before patient implementation and (2) to establish feasibility and refine study procedures prior to recruiting actual patients.

Participants were recruited through email announcements sent to hospital faculty, staff, researchers, and students. Interested volunteers contacted the research team via email and were provided with study information. Inclusion criteria were (1) adult health care workers (aged ≥18 years), (2) proficiency in English, and (3) access to a computer or mobile device with internet connectivity. Exclusion criteria included (1) prior involvement in postoperative instruction material development and (2) visual or hearing impairments that would prevent video viewing or text reading. All instruments were administered online using Google Forms in a single session. Participants accessed the form through a unique link and completed all sections sequentially.

### Ethical Considerations

This study was reviewed and approved by the Mayo Clinic Institutional Review Board (application 25-002248). The study was conducted in accordance with the ethical standards of the responsible institutional committee on human experimentation and with the World Medical Association’s Declaration of Helsinki.

All participants were adult health care worker volunteers (faculty, staff, researchers, and students) recruited through institutional email announcements. Written informed consent was obtained electronically through the Google Forms platform before randomization and data collection began. Participants were informed of the study’s purpose and voluntary nature and their right to withdraw at any time without consequences.

Participant privacy and confidentiality were maintained throughout the study. All survey responses were collected anonymously via Google Forms; no personally identifiable information (eg, name, employee ID, email address, or date of birth) was linked to individual survey responses. Deidentified study data were stored on password-protected, institutionally managed servers accessible only to authorized members of the research team. No images, audio, or video of participants were collected. Only aggregate, deidentified data are reported in this manuscript.

Participants received no financial compensation, gift cards, or other incentives for their participation in this study.

### Educational Materials

#### Text-Based Instructions

We compiled a set of 10 standard postoperative instruction handouts covering common topics relevant to general surgery recovery. These were developed based on established postoperative care guidelines and publicly available patient education resources from major medical institutions [26] and included the following 10 frequently asked topics: pain, pain medication, alarm signs after surgery, surgical drain management, diet after surgery, postoperative nausea, follow-up appointment instructions, recovery instructions, scars after surgery, and suture management [27-36]. Each handout was approximately 100 to 200 words in length and written at an eighth- to ninth-grade reading level [37]. These served as the standard care educational materials for the control group.

#### AI-Generated Video Instructions

**Figure 1.**
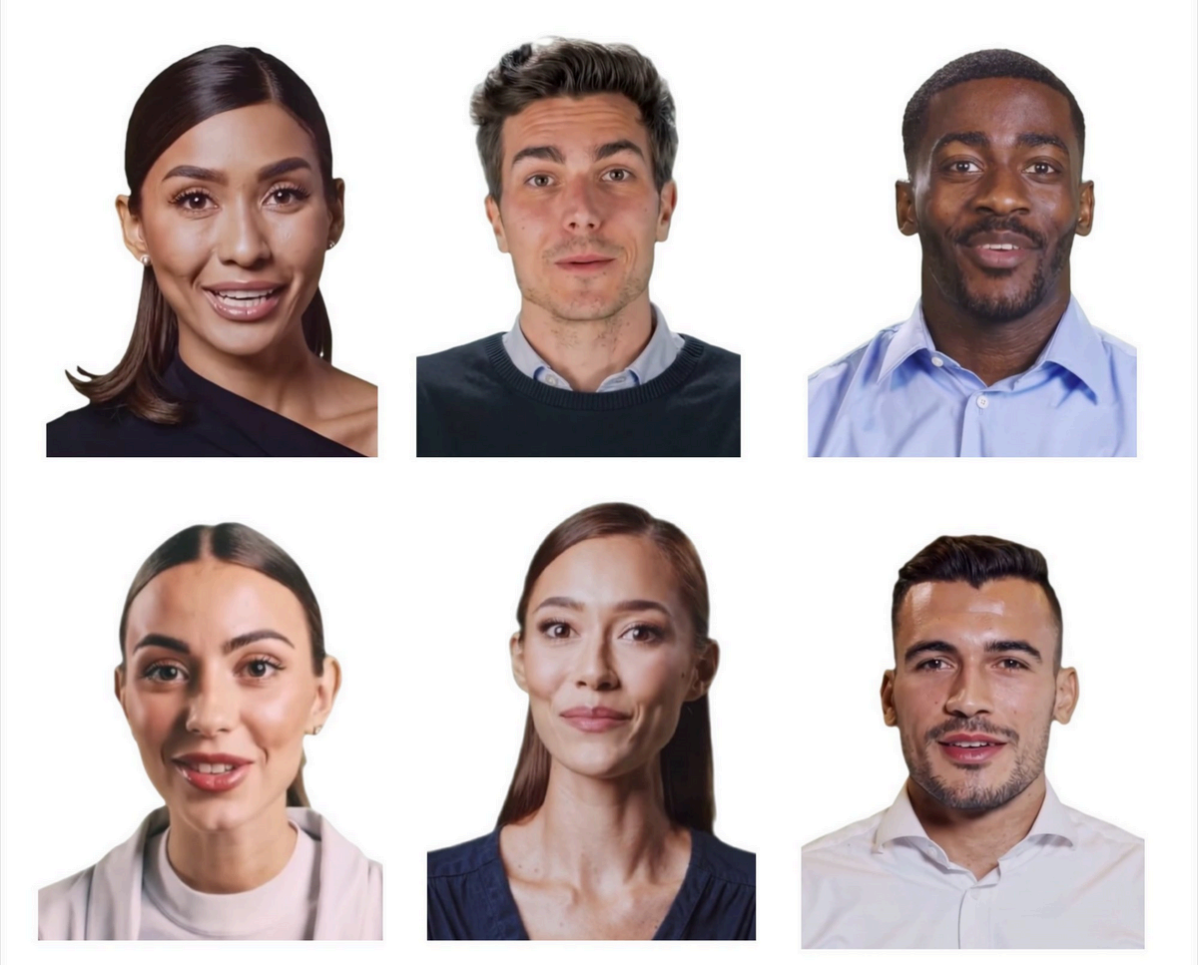
Examples of text-to-video avatars demonstrating the generative artificial intelligence’s ability to generate realistic and diverse human-like representations. Images were generated using HeyGen (HeyGen Inc). Collage created with BioRender.com [39].

Using HeyGen (HeyGen Inc) [38], we created video versions covering the same topics. Scripts were based on the text handout content and edited for a conversational tone. Average video duration was 59.2 (range 45‐84) seconds. We used realistic, diverse avatar presenters (3 male and 3 female avatars) with AI-generated voices. The pain and pain medication topics were merged, yielding 9 videos covering 10 topics. Videos featured avatars speaking directly to viewers, with bullet points and subtitles displayed (Figure 1 [25]).

From a practical standpoint, the HeyGen platform enabled rapid video production without specialized equipment, studios, or video editing expertise. Each video was generated within minutes of script input, and the total time to produce all 9 videos was approximately 2 to 3 hours, including script preparation and quality review.

### Randomization

**Figure 2.**
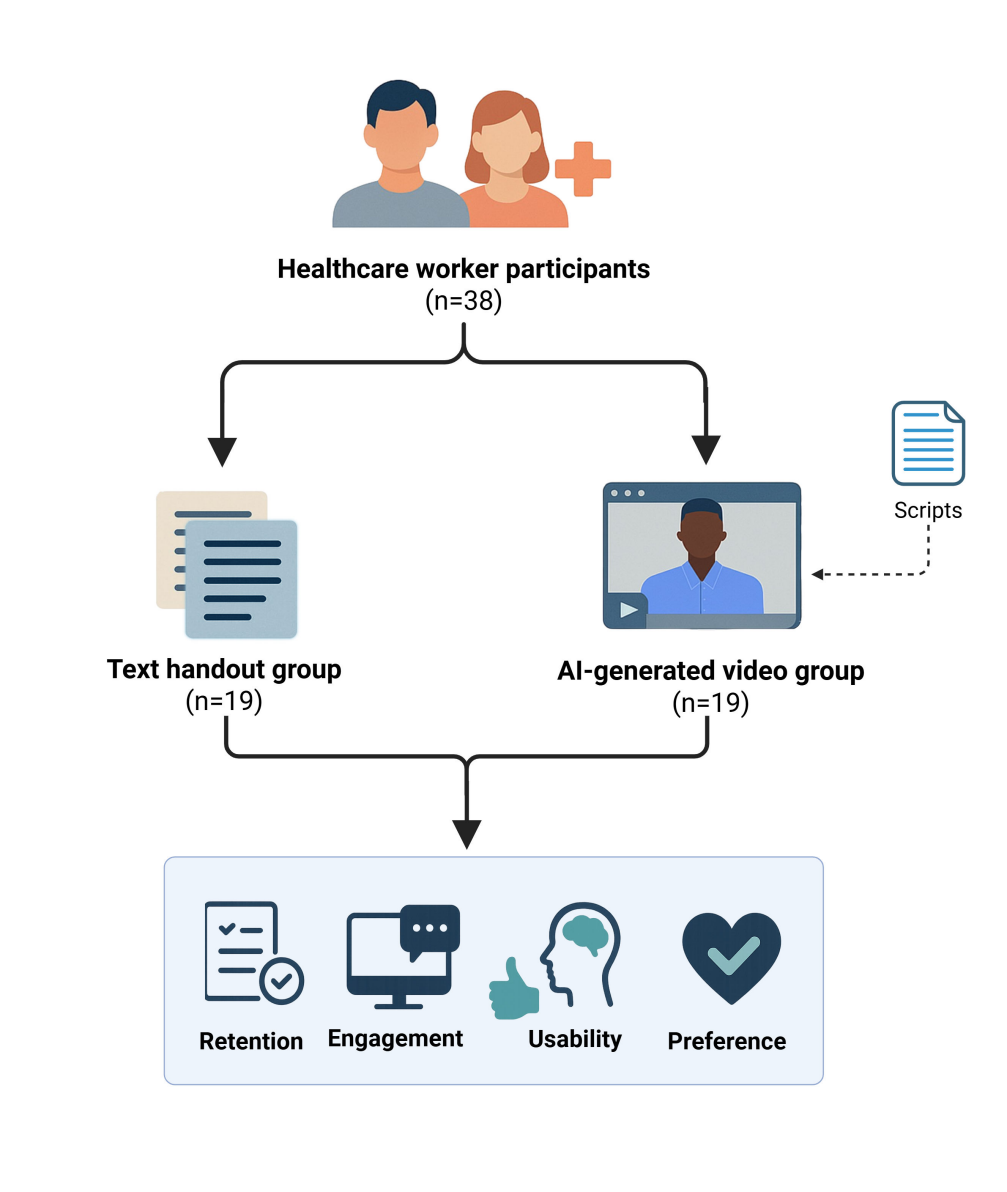
Study workflow demonstrating the allocation of health care workers (HCWs) into 2 groups and subsequent measured outcomes. Image created with BioRender [40]. AI: artificial intelligence.

Volunteers were randomly assigned in a 1:1 ratio to either the text handout group (control) or the AI-generated video group (intervention) using simple randomization (drawing lots). Randomization was performed after informed consent was obtained and before participants accessed the educational materials (Figure 2).

### Outcomes and Measures

Three custom instruments were administered: (1) a 10-item multiple-choice postoperative knowledge assessment quiz (score range 0‐10); (2) a user experience survey with seven 5-point Likert scale items measuring clarity, usefulness, engagement, confidence, retention, preference, and effectiveness; and (3) an engagement metrics questionnaire capturing time spent, materials reviewed, and 3 open-ended questions. Demographics collected included age, sex, education, postoperative care experience, and AI familiarity. Complete instruments are provided in Multimedia Appendix 1. The knowledge assessment quiz was developed by the study team, informed by publicly available postoperative care resources from the American College of Surgeons [26] and Mayo Clinic patient education materials [27-36]. Content validity was established through review by 3 research fellows holding a doctor of medicine or bachelor of medicine and bachelor of surgery degree under the guidance of a plastic surgeon. Educational materials were written at an eighth- to ninth-grade reading level, consistent with recommendations from the National Institutes of Health that patient education materials do not exceed the eighth-grade level [41].

Participants self-reported time spent reviewing materials. Following exposure to the educational content, participants completed the knowledge quiz and user experience survey.

Usability was operationalized through perceived usefulness (“The information provided was useful”), perceived clarity (“The instructions were clear and easy to understand”), and preference for clinical use (“I would prefer video-based instructions over text-based instructions in clinical practice”).

Engagement time and material consumption were self-reported, consistent with standard educational research practices, to avoid technical barriers that automated tracking might introduce.

Qualitative responses were analyzed using a basic grounded theory approach, which is appropriate for exploratory pilot studies seeking to identify emergent themes without predetermined coding frameworks. Two investigators independently reviewed all open-ended responses and conducted open coding to identify recurring themes and sentiments (eg, “video was more personal,” “text was hard to read,” and “avatar speech seemed unnatural”). The investigators then met to discuss their independent findings, reconcile any discrepancies through consensus, and develop a final thematic framework organized into 3 categories: positive aspects, areas for improvement, and clinical practice preferences. Given the limited qualitative data (3 open-ended questions from 38 participants) and the exploratory pilot nature of the study, formal qualitative coding software, interrater reliability calculation, or thematic saturation assessment were not used. However, trustworthiness was enhanced through dual independent coding, consensus meetings, and inclusion of representative participant quotes to support identified themes. All qualitative data were collected and analyzed after completion of data collection.

### Statistical Analysis

Statistical analyses were performed using Python (version 3.12; Python Software Foundation) with the SciPy library. Independent samples 2-tailed *t* tests were used to compare mean scores between groups for all quantitative outcomes. The use of *t* tests for 5-point Likert scales is supported by evidence that parametric tests are robust to violations of normality assumptions, particularly with balanced group sizes [42]. A significance level of *P*<.05 (2-tailed) was used, with effect sizes interpreted as small (0.2), medium (0.5), or large (≥0.8). Effect sizes were calculated using Cohen *d*. Data are presented as mean (SD). Given the pilot nature of the study and multiple comparisons across secondary outcomes, results should be interpreted with appropriate caution.

As this was a pilot study designed to assess feasibility, a formal a priori power analysis was not conducted. The sample size (N=38; n=19 per group) was determined based on feasibility and resource constraints typical of pilot investigations. The observed effect sizes (Cohen *d*=0.45 for knowledge; Cohen *d*=0.81-1.38 for subjective outcomes) can inform sample size calculations for future definitive trials.

## Results

### Participant Characteristics

**Figure 3.**
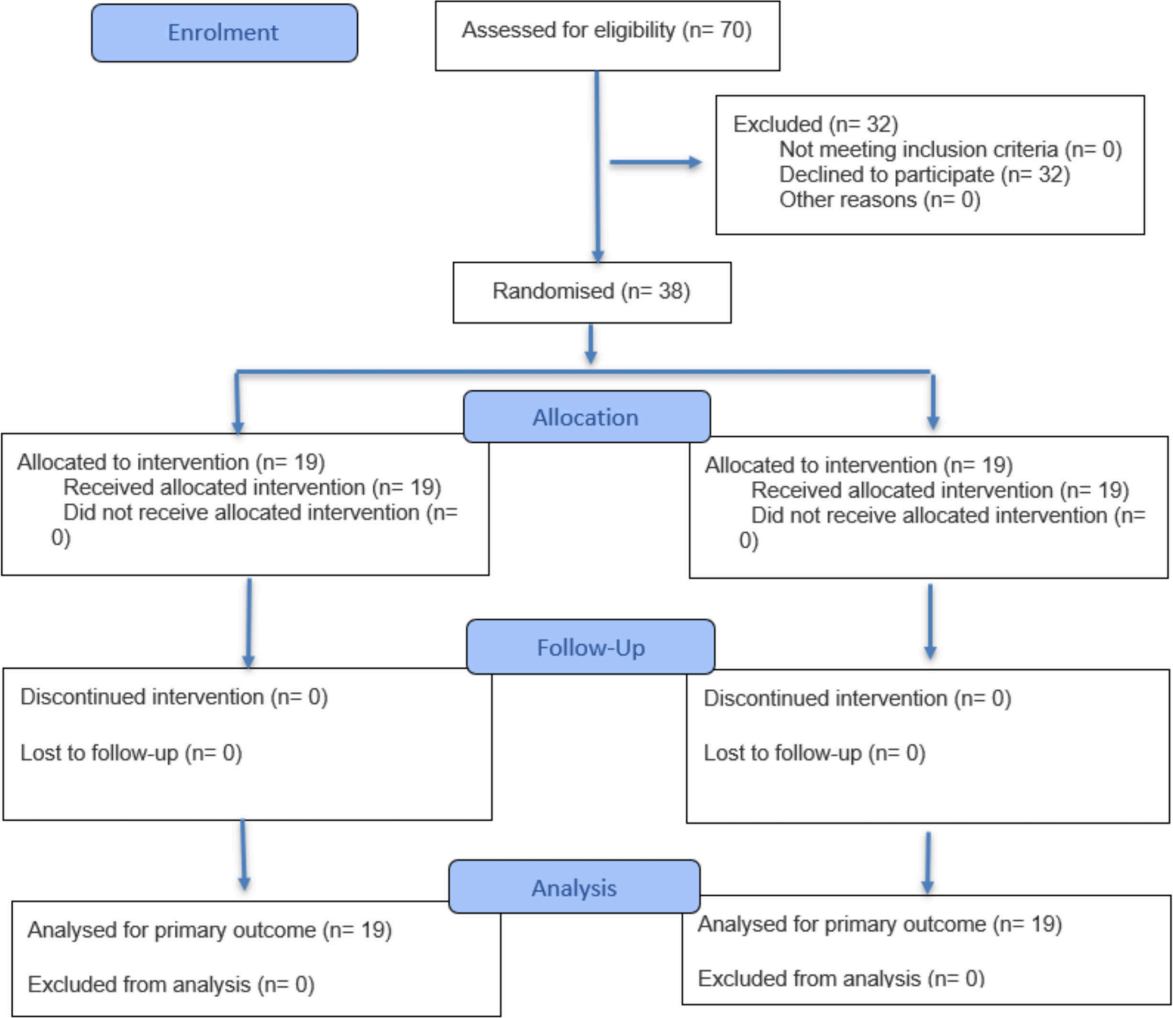
Flow diagram of the progress through the phases of a randomized trial of two groups (that is, enrollment, intervention allocation, follow-up, and data analysis).

A total of 38 volunteers participated in the study, with 19 (50%) participants randomized to the text-based instruction group and 19 (50%) to the AI-generated video instruction group. Table 1 demonstrates that the text handout and AI-generated video groups exhibited similar demographic profiles. The gender distribution was nearly even across both groups, with a slight female majority in both groups. The Consolidated Standards of Reporting Trials of Electronic and Mobile Health Applications and Online Telehealth (CONSORT-eHEALTH) diagram is provided in Figure 3.

**Table 1.** Demographic characteristics of study participants.

Characteristics	Text handout group (n=19)	AI^a^-generated video group (n=19)
Sex, n (%)		
Male	7 (37)	8 (42)
Female	12 (63)	11 (58)
Age (years), mean (SD)	32.53 (6.93)	31.95 (7.68)
Educational level, n (%)		
College (1-4 years)	6 (32)	5 (26)
Graduate (5-7 years)	7 (37)	10 (53)
Postgraduate (>7 years)	6 (32)	4 (21)
Previous experience with postoperative care, n (%)		
Yes	11 (58)	11 (58)
No	6 (32)	6 (32)
Maybe	2 (10)	2 (10)
Familiarity with AI-generated content, n (%)		
Very unfamiliar	4 (21)	4 (21)
Unfamiliar	2 (10)	1 (5)
Neutral	4 (21)	4 (21)
Familiar	5 (26)	4 (21)
Very familiar	4 (21)	6 (32)

a AI: artificial intelligence.

The average age of participants was comparable, with the text handout group having a mean age of 32.53 (SD 6.93) years and the AI-generated video group having a mean age of 31.95 (SD 7.68) years. Educational backgrounds were also similar, with a predominance of graduate-level education in both groups. Regarding prior experience with postoperative care, most participants reported some level of familiarity, though a notable portion, approximately one-third in each group, reported no experience. Familiarity with AI-generated content was also similarly distributed, with 29% (n=11) of participants indicating unfamiliarity, 21% (n=8) expressing neutrality, and 50% (n=19) reporting familiarity.

### Objective Knowledge Assessment

Participants in both groups completed the same 10-item knowledge quiz assessing comprehension of postoperative topics. The AI-generated video group achieved a mean score of 8.89 (SD 1.20) out of 10, while the text handout group achieved a mean score of 8.21 (SD 1.78). This difference was not statistically significant (*P*=.17; Cohen *d*=0.45). Both groups demonstrated high overall comprehension, with mean scores exceeding 80% correct.

### Engagement Levels and Time Spent

Mean completion time for the entire survey (including time spent reviewing educational materials) was approximately 25 minutes for the text handout group and 30 minutes for the AI-generated video group.

Engagement time differed significantly between the groups (*P*=.004; Cohen *d*=1.04). Participants in the AI-generated video group spent more time, on average, engaging with the instructional material than those in the text handout group. The AI-generated video group had a mean engagement time of 15.11 (SD 7.78) minutes, whereas the text handout group’s mean was 8.84 (SD 4.03) minutes (*P*=.004; Cohen *d*=1.04). This large effect size (Cohen *d*>0.8) indicates a substantial practical difference in engagement time between formats. Participants in the AI-generated video group watched, on average, 7.05 (SD 2.67) videos. In contrast, participants in the text handout group read, on average, 6.68 (SD 2.92) instructions. The increased engagement time in the AI-generated video group may reflect participants pausing the videos for various reasons, including reviewing specific information, allowing personal processing time, or taking breaks.

Subjective engagement ratings also differed significantly between the groups. The AI-generated video group’s mean engagement rating was 4.05 (SD 0.78), while the text handout group’s mean engagement rating was 3.32 (SD 1.00; *P=*.02; Cohen *d=*0.81). This large effect size suggests that participants found the video format substantially more engaging than the text format.

### Usability, Understanding, and Retention

In summary, the AI-generated video group demonstrated significantly higher engagement time, perceived clarity, subjective engagement, and information retention than the text handout group. No significant differences were observed in objective knowledge scores, perceived usefulness, confidence in understanding, preference for video-based instructions, or effectiveness in answering postoperative questions.

**Figure 4.**
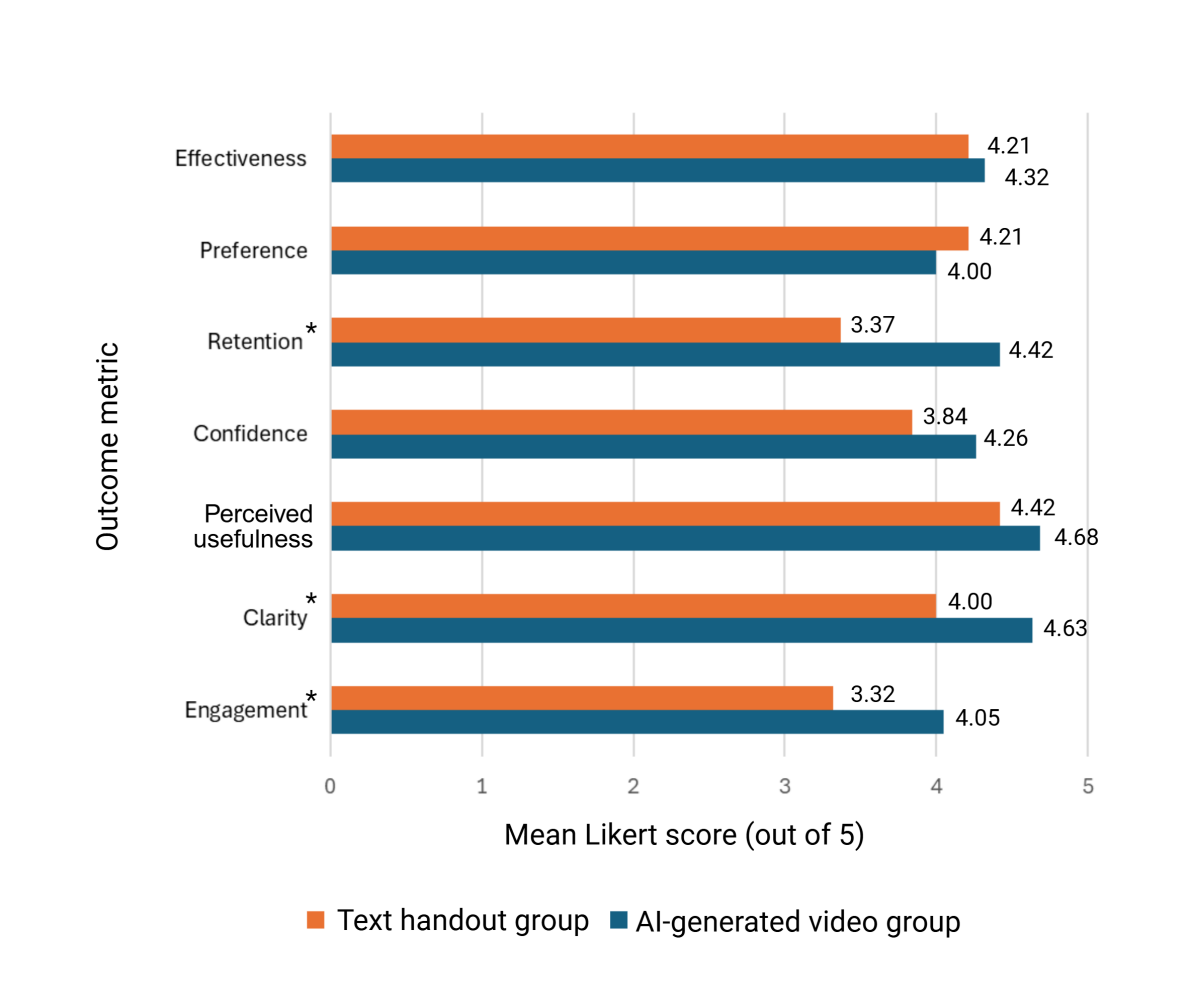
Comparison of text handout and artificial intelligence (AI)–generated video performance across 7 metrics, measured on 5-point Likert scales. The chart displays mean ratings for both groups. *Statistically significant differences (clarity, *P*=.007; engagement, *P*=.02; retention, *P*<.001).

Participants rated the clarity of instructions significantly higher in the AI-generated video group, indicating that they found video instructions clearer than text instructions. Specifically, on a 5-point Likert scale, the AI-generated video group reported a mean clarity score of 4.63 (SD 0.50), while the text handout group reported a mean of 4.00 (SD 0.82; *P=.*007; Cohen *d=*0.93). The large effect size (Cohen *d*=0.93) demonstrates a meaningful practical difference in perceived clarity. However, participants did not perceive a significant difference in the usefulness of the information between the 2 groups. The AI-generated video group reported a mean usefulness score of 4.68 (SD 0.58), and the text handout group reported a mean of 4.42 (SD 0.61; *P=*.18; Cohen *d=*0.43). Figure 4 provides a visual comparison of all Likert scale metrics between the text and AI-generated video groups.

Although participants in the AI-generated video group reported slightly higher confidence in understanding the instructions (mean 4.26, SD 0.65) than the text handout group (mean 3.84, SD 0.83), this difference did not reach statistical significance (*P=*.09*;* Cohen *d=*0.57). Participants found the video format significantly more effective for information retention. They reported a mean Likert retention score of 4.42 in the AI-generated video group, compared to 3.37 in the text handout group (*P<.*001*;* Cohen *d=*1.38). This very large effect size (Cohen *d*=1.38) represents one of the strongest practical differences observed in the study, indicating that participants perceived substantially better information retention with the video format.

Both groups reported similar preferences for video-based instructions in clinical practice, with the AI-generated video group scoring a mean of 4.00 (SD 1.25) and the text handout group scoring a mean of 4.21 (SD 0.71; *P=*.53; Cohen *d=−*0.21). Finally, participants rated the effectiveness of the instructions in answering common postoperative questions similarly across both groups, with the AI-generated video group scoring a mean of 4.32 (SD 0.89) and the text handout group scoring a mean of 4.21 (SD 0.98; *P=*.73; Cohen *d=*0.12). Table 2 tabulates the engagement, usability, and retention metrics between the text handout and AI-generated video formats.

**Table 2.** Comparative analysis of engagement, usability, and retention outcomes between artificial intelligence (AI)–generated video and text instructional formats.

Outcomes	AI-generated video group, mean (SD)	Text handout group, mean (SD)	*P* value
Knowledge quiz score (0-10)	8.89 (1.20)	8.21 (1.78)	.17
Engagement time (minutes)	15.11 (7.78)	8.84 (4.03)	.004
Subjective engagement rating (0-5)	4.05 (0.78)	3.32 (1.00)	.02
Clarity of instructions (0-5)	4.63 (0.50)	4.00 (0.82)	.007
Perceived usefulness of information (0-5)	4.68 (0.58)	4.42 (0.61)	.18
Confidence in understanding (0-5)	4.26 (0.65)	3.84 (0.83)	.09
Retention score (0-5)	4.42 (0.84)	3.37 (0.68)	<.001
Preference in clinical practice (0-5)	4.00 (1.25)	4.21 (0.71)	.53
Number of videos or instructions	7.05 (2.67)	6.68 (2.92)	N/A^a^
Effectiveness in answering questions (0-5)	4.32 (0.89)	4.21 (0.98)	.73

a N/A: not applicable. A P value was not calculated because videos and written instructions are different units of content and cannot be directly compared.

The large effect sizes observed for engagement, clarity, and retention outcomes indicate that the differences between video and text formats represent substantial practical significance beyond statistical significance alone. The moderate effect size for confidence in understanding (Cohen *d*=0.57) suggests a meaningful trend despite not reaching statistical significance (*P*=.09), likely due to our small sample size. In contrast, usefulness, clinical practice preference, and effectiveness in answering questions showed minimal practical differences between formats.

### Qualitative Feedback

Participants provided qualitative feedback on clarity, engagement, suggested improvements, clinical practice use, and format preference. Representative participant quotes illustrating these themes are provided in Table 3.

**Table 3.** Qualitative feedback themes by group.

Themes	AI-generated video group	Text handout group
Clarity	“Crystal clear instructions” and “Clear and direct communication”	“Crisp and clear instructions” and “Clear and easy, no issues”
Engagement	“Engaging like a real guide” and “Lifelike, memorable instructions”	“Quick,” “Not engaging,” and “Less engaging if someone doesn’t like reading”
Suggested improvements	“Gestures not synced with voice,” “Unnatural facial expressions,” and “Slightly robotic voice”	“Could include diagrams or video,” “More visuals or a short summary,” and “Add an FAQ [frequently asked question]."
Clinical practice use	13 out of 19 participants would use; “We are fully engaging when watching videos”	10 out of 19 participants would use; “I’d still prefer a short video to keep people more engaged”
Format preference	“Engaging,” “Easy-to-follow narration,” and “Effective AI explanations”	“Straight to the point” and “Easy to skim”; many preferred video as a supplement

## Discussion

### Principal Findings

This pilot study assessed the feasibility and user experience of AI-generated avatar videos for postoperative instruction delivery among health care workers. The AI-generated video group demonstrated significantly higher engagement, perceived clarity, and information retention than the text handout group, while objective knowledge scores did not differ significantly between groups. These findings suggest that AI-generated avatar videos are feasible to produce using commercially available platforms and may offer meaningful advantages in learner engagement, although their impact on objective knowledge acquisition requires further investigation in patient populations.

The significantly higher engagement with AI-generated videos aligns with media richness theory [42], which suggests that richer media formats are more effective in maintaining attention. The increased engagement time may reflect participants pausing or revisiting video segments to reinforce key points. The superior clarity ratings for video instructions may reflect reduced cognitive load through combined visual and auditory channels [43]. Cognitive load theory suggests that well-designed multimedia can prevent working memory overload, and the conversational tone of AI-generated avatars may create a more approachable instructional style than formal text [13,44].

Participants perceived greater retention with video instructions, consistent with dual-coding theory [45], which posits that information presented in both visual and auditory formats results in more robust encoding. A hybrid approach combining text clarity with video engagement benefits may offer the greatest educational impact [31,32]. The discrepancy between perceived and actual learning outcomes is consistent with existing research [46], suggesting that engagement benefits may not directly translate to measurable knowledge gains in populations with high baseline comprehension.

Qualitative feedback reinforced the quantitative findings, with participants noting the engaging and personal qualities of avatar-led instruction while identifying areas for technological improvement, particularly in avatar speech naturalness and gesture synchronization. These insights will guide iterative refinement of the AI-generated video format for patient-facing deployment.

Several recent studies support the growing viability of AI-generated avatar–based education in health care. Kim et al [47] found that 64% of participants preferred an AI-generated video bot over a text-based chatbot for surgical education. Coleman et al [48] demonstrated that AI-generated digital clinicians achieved significantly higher knowledge scores in a randomized controlled trial. Artsi et al [49] found that while evidence remains sparse, studies consistently report engagement benefits with AI-generated video content. Our findings align with this emerging literature, supporting the engagement advantages of AI-generated video while highlighting the need for validated knowledge assessments in future studies.

### Limitations

Several limitations of our study must be considered when interpreting the results. First, this was a pilot study with a relatively small sample size (N=38), limiting statistical power and the generalizability of our findings to broader patient populations. Participants consisted exclusively of health care worker volunteers (faculty, staff, researchers, and students), and specific clinical roles (eg, physicians, nurses, and administrative staff) were not systematically recorded. Health care workers likely possess higher baseline knowledge about postoperative care than typical surgical patients, which may explain the high quiz scores in both groups (>80%) and the lack of a significant difference between formats. This ceiling effect may have obscured the potential benefits of video instruction that could emerge in populations with lower health literacy. Future studies should include patients with diverse educational backgrounds and health literacy levels.

Second, we used a convenience sampling approach within a single institution, which may have introduced selection bias and further limited the applicability of our results to diverse clinical settings or populations. Additionally, the study evaluated only short-term comprehension and perceived retention, without assessing long-term retention, behavioral adherence, or clinical outcomes such as postoperative recovery or complication rates.

Third, many outcomes, including engagement time, retention, and clarity, were based on self-reported data, introducing the possibility of recall, reporting, or social desirability biases. Objective measures beyond immediate quiz performance were not included, limiting conclusions about true knowledge retention or clinical impact. The observed benefits in engagement and perceived effectiveness may have been influenced by the novelty effect of interacting with AI-generated avatars, which could diminish over repeated exposures or extended use. The longer engagement times observed in the AI-generated video group may have multiple explanations beyond genuine engagement, including time spent adapting to the video interface, pausing to process unfamiliar avatar presentations, or technical factors such as video buffering or lag reported by some participants. These confounding factors limit the interpretation of engagement time as a pure measure of educational engagement. The study also evaluated only a single generative AI platform (HeyGen), and results might differ substantially using alternative AI technologies or avatar presentations.

Moreover, participants reviewed educational materials outside a clinical context, potentially impacting their engagement and motivation differently compared with how real postoperative patients would experience. Furthermore, participants’ technical familiarity and comfort with AI technology varied, possibly biasing results toward those more comfortable interacting with digital or AI-generated content. Finally, our study did not formally assess participants’ baseline health literacy levels, limiting insights into whether the observed improvements are equally beneficial across diverse literacy groups.

Additionally, health care workers do not approximate the general patient population: most participants had college or graduate-level education, were relatively young, and many had prior postoperative care experience. The knowledge quiz was developed by the study team using publicly available patient education resources rather than a validated instrument, and no formal psychometric testing (eg, Cronbach α) was conducted. The multiple-choice format with easily excludable distractors may have allowed correct answers to be guessed through commonsense elimination, contributing to the ceiling effect observed in both groups (>80% correct). The reading level of the educational materials (eighth to ninth grade) exceeds the American Medical Association recommendation of sixth-grade level for general patient populations, although it is consistent with the National Institutes of Health recommendation of no higher than the eighth-grade level [41] and was appropriate for our health care worker sample.

### Future Directions

**Figure 5.**
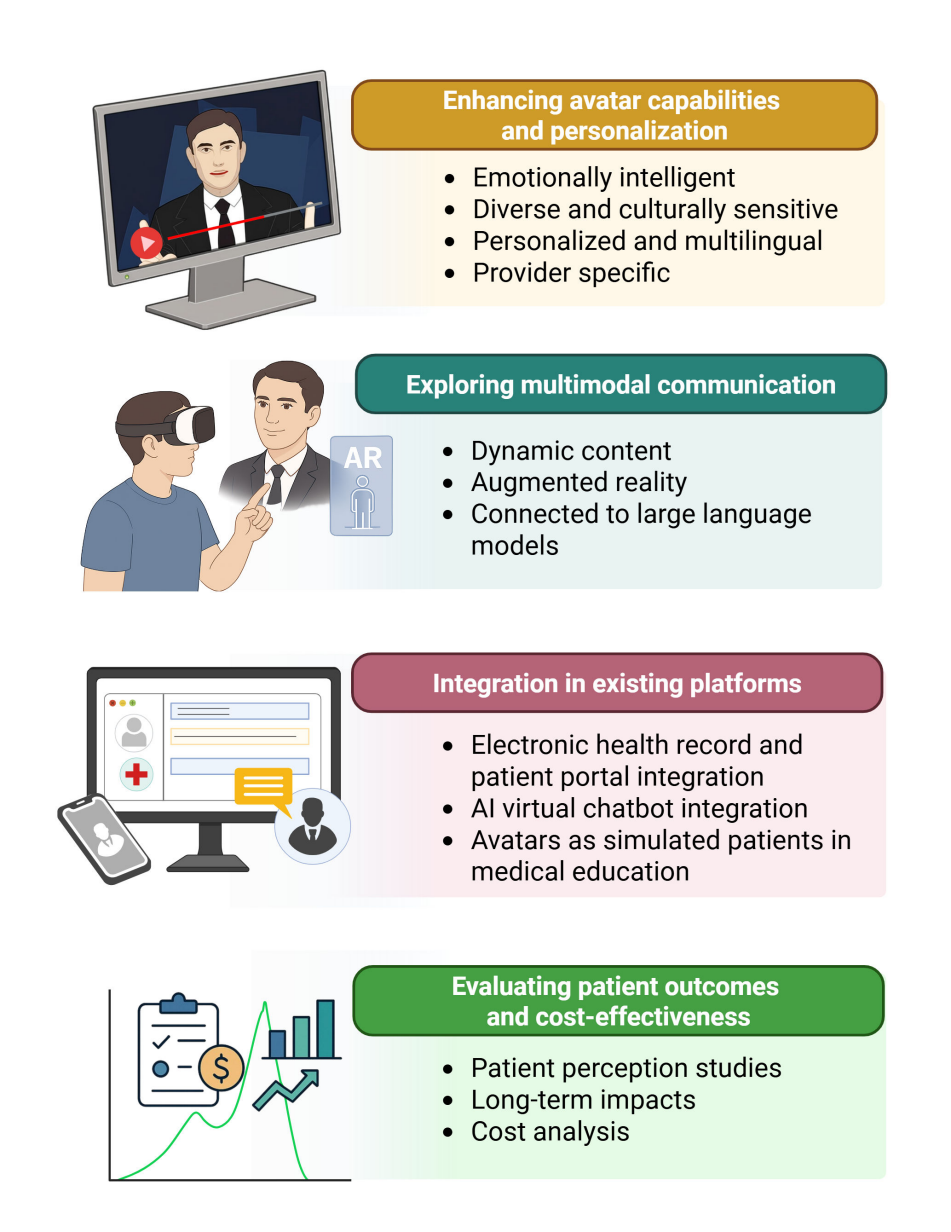
Future directions and recommendations for the use of generative artificial intelligence (AI)–based text-to-video models in health care. Created using BioRender [50].

Future research should evaluate AI-generated video instructions in actual patient populations with diverse health literacy levels. Key priorities include (1) larger randomized trials in surgical patients, (2) assessment of long-term retention and clinical outcomes (eg, adherence and complications), (3) comparison across different AI platforms, and (4) cost-effectiveness analyses comparing AI-generated videos to traditional production methods. Hybrid text-video approaches and culturally tailored avatar presentations warrant investigation [24] (Figure 5 [25]).

Future research should explore multimodal communication, including avatars with dynamic visual aids, augmented reality or virtual reality integration [51], and enhanced natural language processing capabilities through large language model integration. Longitudinal studies assessing sustained educational impact, comparative effectiveness against traditional methods, and cost-effectiveness analyses are needed. Technical priorities include electronic health record integration, 24×7 chatbot availability, and improved accessibility features. Beyond patient education, AI-generated avatars may serve as valuable training tools for medical students in simulated clinical scenarios.

### Ethical Implications of AI

AI-generated avatars raise critical ethical concerns. Transparency is essential—patients must be informed that they are interacting with AI rather than humans to preserve trust and prevent unrealistic expectations. Accuracy requires ongoing monitoring and human oversight, as technological limitations may cause mispronunciations or misrepresentations that compromise comprehension.

Avatar-driven education should complement rather than replace human interaction, as AI cannot authentically replicate empathy and emotional responsiveness. Some users may form unintended emotional connections with AI-generated characters [52,53]; continuous availability of human support can mitigate this risk.

Digital avatars of real individuals require explicit consent regarding creation, distribution, and use. Unauthorized “deepfake” avatars pose significant risks, including misinformation, reputational damage, and erosion of trust [54,55]. Future implementations involving patient data will require robust cybersecurity and privacy protections.

Comprehensive regulatory frameworks addressing consent, ownership, transparency, privacy, accuracy standards, and accountability are essential for the responsible integration of AI-generated avatars in health care [56].

### Conclusions

This pilot study demonstrated the feasibility of rapidly producing AI-generated avatar videos for postoperative instruction using commercially available platforms. Although objective knowledge scores did not differ significantly between formats, the AI-generated video group showed significantly higher engagement, perceived clarity, and information retention. Key limitations include the small sample size, use of health care workers rather than actual patients, reliance on self-reported engagement, and use of a nonvalidated knowledge instrument with ceiling effects. Subsequent evaluation with actual surgical patients has demonstrated high trust and usability for AI physician avatars [25]. Future research should prioritize evaluation in diverse patient populations with varying health literacy levels, using validated instruments and clinical outcome measures.

## Supplementary material

10.2196/89277 Multimedia Appendix 1 Study instruments: 10-item postoperative knowledge quiz, 7-item user experience survey (5-point Likert scale), and engagement metrics questionnaire administered to participants in both the text handout group and the artificial intelligence–generated video group.

10.2196/89277 Checklist 1

## Abbreviations

AI: artificial intelligence
CONSORT-eHEALTH: Consolidated Standards of Reporting Trials of Electronic and Mobile Health Applications and Online Telehealth
