# Supplementary material for: AI-Generated Avatar Videos for Postoperative Patient Education Among Health Care Workers: Pilot Randomized Controlled Trial
Source: JMIR Perioper Med. 2026 May 27;9:e89277. doi: 10.2196/89277 (PMC13215632; doi:10.2196/89277)
Supplement: Multimedia Appendix 1 [file periop-v9-e89277-s001.docx]

**Supplementary Table 1**

**Complete Study Instruments**

*All instruments were administered electronically via Google Forms in a single session. The complete questionnaire as presented to participants is shown below.*

**SECTION 1: DEMOGRAPHICS**

| **Question** | **Response Options** |
| --- | --- |
| Age (Years)* | *Free text (numeric entry)* |
| Gender* | Male / Female / Other / Prefer not to say |
| Education Background* | Primary/basic/elementary / Secondary/middle/intermediate / Tertiary/baccalaureate/high school / Post high school business or trade school / College (1-4 years) / Graduate education (5-7 years) / Postgraduate (7+ years) |
| Previous Experience with Postoperative Care* | Yes / No / Maybe |
| Familiarity with AI-generated content?* | 5-point scale: 1 = Not Experienced 2 = Slightly Experienced 3 = Moderately Experienced 4 = Experienced 5 = Proficient |

**SECTION 2: ENGAGEMENT METRICS**

| **Question** | **Response Format** |
| --- | --- |
| How much time did you spend engaging with the video? (minutes)* | *Free text (numeric entry)* |
| How many videos did you watch?* | Scale: 1–9 (single selection) |
| How engaged were you while watching the videos?* | 5-point scale: 1 = Not Engaged 2 = Slightly Engaged 3 = Moderately Engaged 4 = Engaged 5 = Very Engaged |

*Note: Text group participants received parallel questions asking about time spent reading and number of handouts read.*

**SECTION 3: COMPREHENSION METRICS**

**Self-Reported Confidence**

| Self-Reported Confidence in Understanding Instructions*  5-point scale: 1 = Not at all confident 2 = Slightly confident 3 = Moderately confident 4 = Confident 5 = Very confident |
| --- |

**Postoperative Knowledge Assessment Quiz (PKAQ)**

*10-item multiple-choice quiz. Scoring: 1 point per correct answer, total score range 0–10.*

| **Q#** | **Question** | **Response Options** |
| --- | --- | --- |
| **Q1** | What is a common cause of nausea after surgery? | A) Drinking too much water B) Eating spicy foods C) Anesthesia and pain medications ✓ D) Exercising too soon |
| **Q2** | When should you contact your doctor regarding nausea? | A) If nausea lasts more than a few minutes B) If vomiting leads to dehydration ✓ C) If you feel slightly dizzy D) If you don't feel hungry |
| **Q3** | Which of the following is NOT a recommended pain management strategy? | A) Taking pain medication on schedule B) Using ice packs to reduce swelling C) Rigorous Exercise ✓ D) Getting adequate rest |
| **Q4** | What is a precaution to take when using narcotic painkillers? | A) Drink coffee to stay alert B) Do not drive or operate heavy machinery ✓ C) Skip doses to avoid dependency D) Stop taking them as soon as pain starts to fade |
| **Q5** | When do most patients start feeling better after surgery? | A) Within a few days, but full healing takes weeks ✓ B) Immediately after surgery C) Only after several months D) There is no standard recovery timeline |
| **Q6** | What is a normal symptom during recovery? | A) Mild swelling and fatigue ✓ B) Sharp, severe pain that worsens every day C) High fever and excessive bleeding D) Complete lack of pain after the first day |
| **Q7** | What is an important step in drain care? | A) Keep the drain site dry and clean ✓ B) Submerge it in water for cleaning C) Remove it if it becomes uncomfortable D) Empty it only once every few days |
| **Q8** | When should non-absorbable sutures typically be removed? | A) The day after surgery B) 2 to 3 weeks after surgery ✓ C) Only if they start falling out on their own D) 6 months after surgery |
| **Q9** | What is a red flag that requires immediate medical attention after surgery? | A) Mild bruising around the incision B) Fever above 101.5 degrees F ✓ C) Feeling tired in the evening D) Mild itching around the sutures |
| **Q10** | When should follow-up visits be scheduled after surgery? | A) Only if complications arise B) Within 1 to 2 weeks after surgery, even if there are no complications ✓ C) Only if there is severe pain or infection D) Follow-up visits are not necessary unless advised by the doctor |

*Note: Correct answers are marked with ✓. These were not indicated to participants during the study.*

**SECTION 4: PERCEIVED USEFULNESS & CLARITY**

**User Experience Survey (UES)**

*5-point Likert scale:
1 = Strongly Disagree, 2 = Disagree, 3 = Neutral, 4 = Agree, 5 = Strongly Agree*

| **Statement** |
| --- |
| 1. The instructions were clear and easy to understand.* |
| 2. The information provided was useful.* |
| 3. The format (video/text) made it easier to retain the information.* |
| 4. I would prefer video-based instructions over text-based instructions in clinical practice.* |
| 5. The instructions answered common postoperative questions effectively.* |

**SECTION 5: QUALITATIVE FEEDBACK**

**Open-Ended Questions:**

What did you like about the instructions?*

*(Free text response)*

What could be improved?

*(Free text response)*

Would you use this format (video/text) in clinical practice? Why or why not?

*(Free text response)*

** Indicates required question. Note: Video group and text group received identical instruments with appropriate format-specific wording (e.g., "videos watched" vs. "handouts read").*
